# Medical and Philosophical Intelligence

**Published:** 1812-09

**Authors:** 


### MEDICAL AND PHILOSOPHICAL INTELLIGENCE.

[*The following Letter was received too late for Insertion in its proper Place.*]

### *To the Editors of the Medical and Physical Journal.*

GENTLEMEN,

HAVING, through the medium of the newspapers and other channels, notified to the subscribers to my new work, TYROCINIUM MEDICUM, and to the public, that it would be ready for delivery by the first of August, I consider it incumbent on me to state the reason of the delay in publication, which was solely owing to my waiting to be enabled to give the substance of a new Medicine Act, which obtained the royal assent so lately as the 28th of July last; as I conceived that it would be expected of me to give some account of it, for the information of gentlemen of the medical profession practising pharmacy, (especially in the country,) who might be desirous of knowing how far they may or may not be affected by the clauses of this new act.

It may be remembered that, in the year 1802, I took upon myself to give to the readers of the Medical and Physical Journal, an exposition of an act of parliament, purporting to be an act for the regulation of the duty on quackeries and nostrums; but I found that that act of 1802 did of itself stand in need of so much *explanation* and *amendment*, was fraught with so many dangers to the regular practitioner who did *not* deal in quackery, that, not holding with his lordship the bishop of Rochester's maxim, that "*The people have nothing to do with the laws but to obey them*," I used the freedom to take *the dissecting knife* to said act of parliament, and pointed out its imperfections and dangerous tendency with such effect,* that Parliament granted, in the following year, in consequence of the unwearied diligence and attention of a committee who gave up a great deal of their time towards obtaining the same, a new act, in which the obnoxious clauses of the former act were repealed or ameliorated.

As I had read in the newspapers, in the course of last month, (July,) that *another* new Medicine Act was going through the House of Parliament, I deemed it my duty to watch over the interests of my professional brethren, and to inform them, as soon as possible, how far practitioners in

---

* See vol. viii. of the Medical and Physical Journal, page 127, (for August 1802.)

pharmacy might be affected thereby. For this purpose I made application to Mr. Hanson, the solicitor of the Stamp-office, (to whom I take this opportunity of publicly returning my best thanks for his politeness, and promptitude in answering my inquiries upon *all* occasions.) From the answer which that gentleman was pleased to return me, I had no reason to apprehend that any great inconveniencies would arise to the profession from the passing of this new act. I, however, waited for its being printed; and, having obtained a copy, I have it in my power to inform the profession, that it goes no further to alter the Medicine Act of 1803, than to remove doubts relative to the sale of *soda-water*; and to make a *soda-water*, and the materials for making it, subject to the *stamp duty*. Its principal feature is a new schedule, containing the names and titles of, in all, between five and six hundred QUACK MEDICINES—(mercy on us!)—and the principal object of the bill, is, as I have said before, to render liable to the stamp duty, *from the first day of September in the present year*, 1812, "*All artificial mineral waters, and all waters impregnated with soda, or mineral alkali, or with carbonic acid gas; and all compositions in a liquid or solid state, to be used for the purpose of compounding or making any of the said waters.*"—Therefore, all who make soda-water to send out of their houses for sale, must take out a *medicine licence*; but medical men, who make it only for their own use, and *not* to send it out of their shops, as an article for sale, are not obliged to take out the medicine licence, but must take care not to suffer it to appear without stamps. The fourth clause of *this act* providing, that it shall not be necessary for any victualler, confectioner, pastry-cook, fruiterer, or other shopkeepers in Great Britain, who shall only *sell* any of the artificial or other waters mentioned in the schedule hereunto annexed, *to be drank in his or her house or shop*, and which shall be actually drank therein, to take out a licence for that purpose, under the provisions of the acts of the 42d and 44th year of his Majesty's reign; provided such waters shall be sold by him or her in bottles with paper covers, wrappers, or labels, duly stamped, and properly and sufficiently pasted, stuck, fastened, or affixed, to the same, in the manner hereinbefore mentioned, any thing in the said acts contained to the contrary notwithstanding.—In other respects this new act of July 1812, does not affect the regular practitioner in any way whatever.

I have the honor to be, Gentlemen,
Your very obedient Servant,
WM. CHAMBERLAINE.

29, *Aylesbury-street*,

*Aug.* 17, 1812.

Geological

*Geological Society.—May* 1, 1812.—A paper, by Dr. M'Culloch (member of the society), "On Bister and other Substances produced in the Distillation of Wood; and on their Analogy with the Native Bitumens," was read.

When wood is submitted to destructive distillation, there is obtained, among other products, a black substance resembling common tar. This tar is very inflammable, and so liquid that it may be burnt in a lamp. By washing it with water, either hot or cold, or submitting it to the action of lime or of the mild alkalies, a large portion of acetic acid is separated, and the residue becomes pitchy and tenacious. It is entirely soluble in caustic alkali, in alcohol, in ether, in acetic acid, and in the mineral acids. The fat oils and the recent essential oils dissolve but little of it; but, if the former are made drying, and if the latter have become brown by keeping, they then act more readily and copiously. Colored oil of turpentine takes up a considerable quantity, but naphtha only acquires a scarcely sensible brown color by digestion upon it. When carefully distilled, at a gentle heat, it is decomposed into an oily matter, at first limpid and afterwards brown, a quantity of acetic acid combined with a little ammonia; and a spongy coal remains in the retort. In this process no inflammable gas is given out; but, at a high temperature, the oil is more or less decomposed, and an inflammable gas is produced, which, however, does not burn with a flame by any means so bright as the gas from pit-coal. If this destructive distillation is not carried very far, the matter in the retort will be found, when cold, to be solid, brilliant, shining, and possessed of a conchoidal fracture: its taste is burning and pungent, and its odour is that of wood smoke; it is fusible, and readily inflammable. When kept melted in an open vessel till it ceases to be fusible, it becomes more and more brilliant, its fracture passes to splintery, and it assumes the perfect appearance of asphaltum. In proportion as it approaches this state it becomes less and less soluble in alcohol, and at length scarcely gives a stain to this menstruum. Naphtha has no action on it, and in this circumstance alone it differs from asphaltum. Dr. M. then proceeds to an examination of the bitumens, and shows that the difference between the products of recent vegetable matter, and the bitumens when subjected to distillation, consists in the former yielding empyreumatic acetic acid and a black pitchy matter insoluble in naphtha; while the latter afford ammonia and naphtha, but little or no acid. He then enters into a detailed investigation of the properties of the very important class of *Lignites*, or those substances, such as peat, surturbrand, Bovey coal, &c. in which the traces of vegetable origin are not obliterated.

Submerged wood from peat mosses gave a brown oil smelling of wood-tar, and refusing to dissolve in naphtha.

A compact pitchy-looking peat gave a fetid oil resembling in odour neither wood-tar nor bitumen, and very slightly soluble in naphtha.

Bovey brown coal gave an oil resembling in odour that of wood-tar, but much more soluble in naphtha: that portion of the oil which was insoluble in this menstruum had a strong odour of wood smoke.

The oil of jet was almost perfectly soluble in naphtha, and smelled strongly of petroleum; but it afforded also empyreumatic acetic acid.

Thus it appears that there exists a class of fossils of undoubted vegetable origin, which exhibit the gradual progress from wood to bitumen, and in which this change has been brought about by the action not of heat but of water.

The experiments, however, of Sir James Hall seem to show that heat, with compression, is also capable of converting wood into coal. —A critical examination of this fact was the next object of Dr. M.; and he found, on heating wood in close gun-barrels, that a black coaly-looking substance was indeed produced, but that it consisted wholly of charcoal, empyreumatic acid, and wood-tar, and did not contain the smallest portion of real bitumen. Hence the experiments alluded to by no means prove the possibility of converting vegetable matter into real coal by mere heat. It appears, however, to Dr. M. that the consolidation of bituminized vegetables into coal is not unlikely to be the effect of subterranean heat.

The paper concludes by showing the identity of the pitch procured from the distillation of wood and the pigment called *Bistre*; and points out methods of obtaining it in a state better fitted than common bistre for the purposes of the artist; and also enumerates several other uses to which this substance may be œconomically applied.

Some Notes on the Mineralogy of the Neighbourhood of St. David's, in Pembrokeshire, by Dr. Kidd, were read.

The country about St. David's, when viewed from an eminence, presents the appearance of an extensive uneven plain, interspersed with numerous detached hills or rocky summits of an irregular conical shape; the two highest of these hills are Penbury and Carn-Llidy, the western portion of the latter of which forms the promontory of St. David's-head.

These hills present no appearance of stratification, and are composed of feldspar and hornblend in various proportions and states of aggregation. They are each surrounded by mantle-shaped strata of slate, elevated at high angle, and presenting the characters of grauwakke-slate. This latter is transversed by veins of quartz, from which very fine specimens of rock-crystal are procured. No carbonate of lime appears to be contained either in the unstratified trap or in the slaty grauwakke; nor did there occur in them, with the exception of one equivocal instance, the smallest trace of any organic remain.

*May* 15.—An Account of the Island of Teneriffe, by the Hon. Henry Grey Bennet, was read.

The greatest length of this island, from north to south, is about seventy miles, its greatest breadth does not exceed thirty miles. In the south-western part of the island is situated the mountain, called by the Spaniards, El Pico di Tiede, but better known by the name of of the Peak of Teneriffe, the height of which, from the mean of several observations, appears to be about 12,500 English feet.

The rocks and strata of this island appear to be wholly volcanic. A long chain of mountains passes through the interior, sloping on the eastern, western, and northern sides of the sea, but on the S. and S.W. elevated into nearly perpendicular mountains, which are intersected by deep and narrow ravines.

The lowest bed of the island is porphyritic lava composed of hornblend and feldspar, in its upper part porous, scoriform, and sometimes passing into the state of pumice. Upon this rests a bed of the same substances as already mentioned, but, in structure, nearly approaching to greenstone. This is covered by a thick bed of pumice, which itself is overspread with basaltic lava, on which, in many places, rests beds of tufa and volcanic ash.

This basaltic lava decomposes sooner than any of the other rocks, and contains the greatest variety of imbedded substances: it is sometimes divided by a layer of olivine in crystals some inches long, and is often intersected by thick veins of porphyritic slate. Zeolite and chalcedony also occur in it.

The number of small craters and extinct volcanoes is prodigious; —they are to be found in all parts of the island, but none of them have been in activity of late years. The great streams of lava have flowed from the peak; those of the years 1704 and 1797 (which was the last) are basaltic. This latter flowed so slowly, notwithstanding the sharp descent of the mountain, that it was several days in advancing three miles. On the south-western side of the peak is an ancient lava not at all decomposed, several miles in length, and in a perfect state of vitrification resembling obsidian.

*June* 5.—"An Account," by Thomas Webster, esq. "of some new Varieties of Alcyonia, found in the Isle of Wight," was read.

In viewing the rocks about Ventnor Cove and in various parts of the undercliff, Mr. Webster remarked, in the sandstone stratum immediately under the chalk-marl, a great number of small prominences resembling in form the branches of trees. They were of various sizes, from half an inch to three or four inches in diameter: their substance was sandstone of the same kind as the rocks they were in; but the part resembling the bark was somewhat harder, which enabled it to endure longer than the rest of the stone, and thus project above its surface. Some of them were straight, others a little crooked, and, in a few instances, he observed them forked. He found fragments of these bodies in every part of the island where the sandstone stratum can be seen, and particularly among the masses of rock lying under the cliffs of Western Lines. In this last place he found that the stems above-described had frequently heads or bulbous terminations attached to them, in form somewhat resembling a closed tulip, and in some of these he found distinct traces of organic structure, from which it appeared that these heads consisted of a group of *tubuli* now converted into and enveloped with stony matter. Besides these extraordinary shapes which projected in relief, Mr. W. observed a variety of very regular white figures as if painted upon the the rock, being even with its surface. They consisted of circles from two inches to half an inch in diameter, ellipses of various eccentricities, and parallel lines both straight and crooked.

By a careful examination, Mr. W. found that these white figures belonged to the other class of bodies already described; that the cylinders were only the internal parts of the same body, whose various sections formed the white circular and elliptical figures. The vast masses of rock which had fallen down, having separated from the cliff at the divisions between the beds, showing their upper and under surfaces covered with layers of these bodies heaped upon each other and lying prostrate in every possible direction; and, in the joints between the beds where they were not separated, they were distinctly seen.

The green sandstone and the limestone he found to be the chief repositories of these bodies. In the ferruginous sand below the green sandstone he found none, and only a few fragments of cylinders in the blue marl on which the sandstone rests. He traced them upwards into the chert; but they there became rare, and they totally disappeared in the chalk-marl.

He found them, however, frequently in the fragments of flint lying on the shore. Mr. Webster having brought away an extensive series of specimens (which he has since deposited in the collection of the Society), submitted them to the examination of Mr. Parkinson, who is of opinion that they belong to the genus Alcyonium, but that they are of three or four different species, neither of which have been hitherto described. From the resemblance which these bodies bear to a closed tulip attached to its stalk, Mr. W. suggests that the name of *Tulip Alcyonium* may not be improperly applied.

"Some Observations," by James Parkinson, esq. "on the Specimens of Hippurites from Sicily," were read.

These specimens Mr. Parkinson considers to be such as demand particular attention, as they possess those characters which will probably serve to correct some erroneous opinions respecting the nature and habits of the animals of which these shells were the dwellings.

One of the specimens contains a nearly perfect shell, longitudinally divided so as to display the two ridges with the numerous septa and chambers.

From an examination of these specimens, and by comparing them with the observations he has before had an opportunity of making, Mr. Parkinson is of opinion that the structure of the shell of the Hippurites is such as would enable the animal to raise itself to the surface of the water.

This opinion is in opposition to that of M. Denys de Montfort and most of the French oryctologists, who consider the Hippurites as belonging to what they term pelagian shells, or such as constantly inhabit the bottom of the sea, never rising to the surface or appearing on the shores; and therefore that there is no reason to believe them as belonging to animals which are now extinct, but only that their recent analogues have not yet been brought to view.

A paper by Joseph Skey, M.D. entitled "Some Remarks upon the the Structure of Barbadoes, as connected with Specimens of its Rocks," was read; together with a Note by Mr. Parkinson, on some of the specimens presented by Dr. Skey.

The island of Barbadoes is totally unlike those immediately near it, both in appearance and in structure. The land rises in a gentle swell from the coast towards the middle of the island, except in one small district. Its highest hills do not exceed 800 or 900 feet, and their general direction is nearly N.W. and S.E. Upon the north-eastern coast the shores are bolder than in the other parts of the island, as is the case in many of the islands of these seas.

Barbadoes is composed of limestone, in great part of fossil madre-pores, and traces of organic structure are to be met within almost every part of the island, more particularly along the whole of the S. and S.W. coast.

The land which when seen from the sea appears to rise uniformly from the coast, is observed on a nearer view to consist of successive terraces rising in two or three gradations, one above the other, each forming a plain of a quarter or half a mile in breadth, and terminated by a cliff of coral rock varying in elevation from twelve to twenty feet, and sometimes considerably higher.

Deep fissures have in many places of the island rent asunder the cliffs, and these gullies (as they are called) are continued across the terraces in irregular lines. Numerous caves are every where to be met with, and they are sometimes of very large dimensions.

On the S. and S.W. side of the island there may be seen at very low water a bed of calcareous sandstone dipping S.W. thirty degrees. To the eastward of the garrison of St. Ann, there is found a dull compact chalky-looking limestone with ramose alcyonia, while considerably to the westward the rock is more distinctly coralloidal.

Upon the northern and north-eastern side of the island is a small mountainous district called Scotland. It consists almost entirely of limestone, but of a kind less marked by organic remains than in the other districts.

In Mr. Parkinson's Note it is observed that some of Dr. Skey's specimens illustrate the nature of some fossil corals; showing that the forms in which they at present exist, are not those which belong to those substances in their original state, and consequently ought not to affect their specific or generic distinctions.

A letter from E. L. Irton, esq. describing some remarkable tubes found in the drifted sand at Drigg, in Lancashire, was read; together with an account, by W. H. Pepys, esq. of a chemical examination made by him of the substance of these tubes.

These tubes are nearly in a perpendicular position, imbedded in the midst of the hills of drifted sand, on the sea shore, without any communication with the surface; and there are ramifications extending from them which generally point downwards, and terminate in fine points. The tube sent to the Society is above an inch in diameter, and of an irregular form: the outside consists of black and white sand agglutinated together; the inside is smooth, and has a vitrified appearance. When dug out of the sand it was soft, and in in some degree flexible; and the inside coating, at its first exposure to the air, was soft to the touch, and rather unctuous; but in less than a quarter of an hour it hardened into the state in which it now exists.

The tube when found was filled with the sand of the hill, and that sand is quite different from the sand of which the outside of the tube consists.

Both the sand and the vitreous part of the tube scratch glass; and on the latter, when viewed by a lens, there are seen small air-blebs, such as are common to imperfect vitrification. Both are insoluble in sulphuric and nitric acids; infusible before the blowpipe without addition, and partly fusible on the addition of boracic acid; but with soda a complete fusion took place, and the residue was nearly soluble in water.

A paper by Dr. M'Culloch, "On the vitrified Fort of Dun Mac Sniochain, near Oban, in Argyleshire," was read.

In the discussion which some time ago took place respecting the vitrified forts of Scotland, the question on which the two contending parties were most at issue, was, whether the vitrification was the effect of design or of accident. It occurred to Dr. M. that light might be thrown on the subject by examining with mineralogical accuracy the substance of which these structures were composed, and noting the changes which each had undergone in consequence of the fire, and also by observing whence the stones had been derived which were used in them; and that the question of accident or design might be illustrated, by examining in the laboratory the degree of heat required to produce the appearances in the stones which actually existed in these structures.

The fort of Dun Mac Sniochain stands on a long narrow hill, which is nearly precipitous along three parts of its circumference, and at the other end it rises from the plain with a very accessible acclivity. The walls, which are all at present buried under the soil, are about eight or ten feet in thickness. They bear marks of vitrification through their whole extent: but in no case does it appear to have extended more than a foot or two upwards, and the most perfect slags are found at the bottom of the foundation. In the higher parts of these are stones roasted by the action of the heat, but unvitrified, and at length the marks of fire almost entirely disappear. The hill consists of alternate beds of schistus and limestone, but the latter is the predominant rock.

It is perfectly insulated in a great alluvial plain. The mountains of Benediraloch, which bound the plain to the west, consist of granite gneiss, mica-slate, quartz, and porphyry. On the edge of these rocks are found large detached masses of puddingstone, consisting of rounded pebbles of greenstone of different varieties, of amygdaloid and quartz cemented by a paste which appears to consist chiefly of trap sand, united by the hard variety of calcareous spar. The paste contains also in small quantity zeolite, prehnite, garnet, and diallage. This puddingstone, where nearest the fort, is at least half a mile distant from it. The walls of the fort consist principally of granite

 gneiss, gneiss, mica-slate, clay-slate quartz, puddingstone, and pyritical slate, entangled together with a very small proportion of the particular rock on which the fort itself is founded; puddingstone forming the greater part of them. This puddingstone Dr. M. shows to be the only vitrifiable ingredient of the walls; and, from the distance from which it must have been brought, and the great quantity of it employed in the work, he considers it probable that the builders of the fort must have been acquainted with its vitrifiable nature, and that it was on account of this quality that they had employed so great labor in transporting it. For, if their object had not been to produce vitrification, but merely to erect a dry wall of stone, the limestone of the hill would have answered their intentions, or perhaps the loose stones of the adjoining plain.

That they did not obtain the puddingstone from the latter source, is evident; for, although the plain and shore are covered with fragments, these consist almost entirely of the primary rocks: and, besides the pieces of the wall which have not felt the fire, there are angular fragments, showing pretty clearly that they were not collected on an alluvial plain, but broken from the rocks where they are found.

Dr. M. next proceeds to describe the various states in which the different stones are found.

The puddingstone exhibits the greatest variety of changes. It is found in every state, from a black glass to a spongy scoria capable of floating in water, sometimes exhibiting the gradual succession of changes from incipient calcination to complete fusion. To ascertain the degree of heat necessary to produce the corresponding changes in this rock, Dr. M. submitted various parts of it to the furnace, and found that some of the fused substances must have been brought to that state in a heat not less than 100° of Wedgwood's scale; a heat at which many varieties of earthen-ware are baked.

Dr. M. next gives a short account of the vitrified fort of Craig Phadric, in Invernessshire, and of another in Galloway; in both of which, but more particularly in the former, he observed circumstances quite analogous to what he had already found at Dun Mac Sniochain; and the conclusion he has been led to form is, that the vitrification of these forts is the effect of design.

---

Messrs. Longman and Co. have nearly ready for publication, Engravings from Specimens of Morbid Parts, preserved in Mr. Charles Bell's collection, Windmill-street, and selected from the divisions inscribed Urethra, Vesica Urin. Morbosa et Læsa; containing specimens of every disease which is attended with change of structure in these parts; and exhibiting the injuries from the bougie, catheter, caustic, trochar and lithotomy knife, incautiously used. The work will be published in four fasciculi of ten plates each, in folio. The first and second fasciculi will contain specimens of diseased urethra, and of the canal injured by operations; with plans to direct the use of instruments.

In the hill about a mile north of Stapleford, in Nottinghamshire, consisting of gravel rock, very similar to that of which Alderley-Edge, N.W. of Macclesfield, in Chester, is composed, but not resting on and surrounded by red marl as that hill does, but on and by coal-measures, a vein of ore of manganese, iron, and copper, with much mica, was lately discovered, on the estate of Admiral Sir John Borlase Warren, bart. who has caused some of the ore to be smelted and the copper refined, which proves to be of the very best quality. Sir John had a coal-seam seven feet thick lately in work, about 300 yards S. of this mine, and has proved the same coal-measures at about 4 or 500 yards N. while Lord Middleton's large collieries, in Trowel, are not more than 800 yards distant, in the same direction.

---

*Vaccine Inoculation.*—At a meeting of the Board of Managers of the London Vaccine Institution, July 30, 1812,—It appears that in the last year there have been vaccinated by Dr. Walker, the resident inoculator, 2303 persons; from the beginning, in 1806, 10,898 persons.—By the appointed inoculators in the metropolis, 1075; from the beginning, 4184.—By the appointed inoculators in the United Kingdom, 36,733; from the beginning, 214,207: making a total of 229,289 persons who have received the protecting benefit of vaccination, under the auspices of this Institution.—It also appears that the resident inoculator has, during the last year, supplied 25,923 charges of vaccine lymph to 5256 applicants, not only in the metropolis, and different parts of the United Kingdom, but to the colonies, the army and navy, and to foreign countries. The total supply of vaccine lymph, afforded by the Institution, amounts to 119,003 charges to 24,156 applicants.

---

A medicated Vapor Bath, after the Turkish plan, has recently been fitted up at No. 5, Downing-street, and promises to be an useful acquisition to medical practitioners, who may wish to employ the bath under their immediate direction.

---

*Theatre of Anatomy.*—The Autumnal course of Lectures on Anatomy, Physiology, Pathology, and Surgery, by Mr. John Taunton, F.A.S. Member of the Royal College of Surgeons of London, Surgeon to the City and Finsbury Dispensaries, City of London Truss Society, &c. will commence on Saturday, October 3, 1812, at eight o'Clock in the Evening *precisely*, and be continued every Tuesday, Thursday, and Saturday, at the same hour.

In this course of Lectures it is proposed to take a comprehensive view of the structure and economy of the living body, and to consider the causes, symptoms, nature, and *treatment of surgical diseases*, with the mode of performing the different surgical operations; forming a complete course of anatomical and physiological instruction for the medical and surgical student, the artist, the professional or private gentleman.

An

An ample field for professional edification will be afforded by the opportunity which pupils may have of attending the clinical and other practice of both the City and Finsbury Dispensaries.

---

Dr. Reid's next course of Lectures on the Theory and Practice of Medicine, will commence at ten o'clock in the morning, on Monday, the 5th of October.

---

Dr. Merriman, Physician-Accoucheur to the Middlesex Hospital, and the Westminster General Dispensary, will begin a course of Lectures on Midwifery and the Diseases of Women and Children, at the Middlesex Hospital, on Monday, October the 12th.

---

Dr. Clutterbuck will begin his Autumnal course of Lectures on the different Branches of Medicine, on Monday, October the 5th, at ten o'clock in the Morning.

The different lectures will be delivered on the alternate days, at the same hour, viz. on the *Theory and Practice of Physic*, on Mondays, Wednesdays, and Fridays; and on *Materia Medica and Chemistry*, on Tuesdays, Thursdays, and Saturdays, throughout the season.

---

Dr. Clough, of Berner's Street, will commence his Autumnal course of Lectures on the Science and Practice of Midwifery, including the Diseases of Women and Infants, as connected therewith, early in October next; in the morning at half past ten, and in the evening at seven; timely notice of which will be given in most of the morning and evening papers.—Students, when properly qualified, will be amply provided with cases to attend in rotation, which are governed by the number admitted at the institution; their views will be greatly forwarded by weekly examinations of the preceding lectures.

---

Dr. Ramsbotham will commence his first Winter course of Lectures on the Science and Practice of Midwifery, and on the Diseases of Women and Infants, on Monday, October 5th, 1812.

The Prospectus may be had of all the medical booksellers.

---

*St. Thomas's and Guy's Hospitals.*—The Winter course of Lectures at these adjoining hospitals will commence as usual on the 1st of October, viz.

At St. Thomas's—Anatomy, and the Operations of Surgery; by Mr. A. Cooper and Mr. Henry Cline.—Principles and Practice of Surgery; by Mr. A. Cooper.

At Guy's—Practice of Medicine; by Dr. Babington and Dr. Curry.—Chemistry; by Dr. Babington, Dr. Marcet, and Mr. Allen. —Experimental Philosophy; by Mr. Allen.—Theory of Medicine, and Materia Medica; by Dr. Curry and Dr. Cholmeley.—Midwifery, and Diseases of Women and Children; by Dr. Haighton.—Physiology, or Laws of the Animal Œconomy; by Dr. Haighton.—Structure and Diseases of the Teeth; by Mr. Fox.

N.B. These several lectures are so arranged, that no two of them interfere in the hours of attendance; and, with the lectures on Anatomy, and those on the Principles and Practice of Surgery, given at the theatre of St. Thomas's Hospital adjoining, the whole is calculated to form a complete course of Medical and Chirurgical Instructions.

---

Mr. Moor, Surgeon-Dentist to Her Royal Highness the Dutchess of York, will commence a course of Lectures on the Structure and Diseases of the Teeth, on the 4th of November, in which will be explained, the complete Practice of the Dentist.

---

*London Hospital.*—Dr. Buxton's Lectures on the Practice of Medicine, will be commenced on Thursday morning, October 1st, at eleven o'clock.

---

*Theatre of Anatomy, Bartlett's Court, Holborn.*—Mr. Armiger will commence his Lectures on Anatomy, Physiology, and Surgery, on the 2nd of November.—The theatre will be opened for Practical Anatomy on the 1st of October, at nine in the morning.

---

Mr. Carpue will commence his Lectures on Anatomy, Surgery, &c. on the 2nd of October.

---

*Dr. Clarke's and Mr. Clarke's Lectures.*—Dr. Clarke and Mr. Clarke will begin their Lectures on Midwifery, and the Diseases of Women and Children, on Monday, October the 5th.

The lectures are read every day, from a quarter past ten o'clock in the morning till a quarter past eleven, for the convenience of students attending the hospitals.—The students will be provided with cases, when they are properly qualified.

---

*On the Composition and use of a new Solution of Ferrum Tartarisatum.*—By George Birkbeck, M.D.—In consequence of some experiments on the preparation and properties of ferrum tartarisatum which were mentioned in the tenth number of the London Medical Review, I have been induced frequently to employ it dissolved in water, in various states of disease. Having found that the medicinal efficacy of the iron was equal if not superior to that of any preparation in common use; and likewise that the ferruginous flavour was much less perceivable than in any other compound of an acid and the same quantity of the metal, I have recommended to several practitioners, its employment instead of vinum ferri, mistura ferri composita, or ferrum vitriolatum. From many of these I have received favourable reports of its operation, and I am therefore desirous through this medium still further to extend the knowledge of it.

When

When water is poured on ferrum tartarisatum prepared according to the imperfect directions contained in the London Pharmacopœia, a portion is dissolved; but as it contains much metallic iron, with very little of the tartrate of iron, a medicine is formed of very trifling power. Even when the ferrum tartarisatum is made according to the judicious directions conveyed in the publication before mentioned, an imperfect although much more powerful solution is obtained. This solution is imperfect because liable to decomposition and change, even if kept in close vessels. In order to obviate this imperfection, Mr. Phillips, who has prepared all the solution with which the trials have hitherto been made, has adopted the following process.* Sixty-four parts of cream of tartar, are to be mixed with thirty-two of filings of soft iron: to the mass during the action of the tartar upon the iron, water should be occasionally added; and the digestion is to be continued until it appears, by the test of litmus paper, that the acid is perfectly saturated with iron. To this seven times its weight of water is to be added, which easily dissolves the tartarised iron, when triturated, and the fluid readily passes through the filtre. For the purpose of saturating any redundant acid, Mr. P. has recently added ammonia, expelling that which may be in excess by subsequent exposure to heat. After standing some time there is a deposit of tartrite of lime from the solution thus prepared, which being removed, it is permanently transparent. The color of the solution is a deep greenish brown, having, when the acid has been perfectly saturated, very little of the chalybeate taste. It contains about one eighth part of its weight of tartarised iron, or about 3·3 per cent. of peroxide; indeed the quantities have lately been so adjusted by Mr. Phillips, that each fluidounce of the solution contains exactly sixteen grains of the oxide.

In exhibiting the solutio ferri tartarisati it may be mixed with plain water, or with any of the aromatic waters. It may also be given along with the tonic and bitter vegetable infusions, and with any of the carbonated alkalies, or even pure ammonia, none of these having the power to decompose the tartrite of iron. In all the cases in which iron is indicated this preparation may be employed; and it seems peculiarly well fitted for those disorders in which the nauseous mixture recommended by Dr. Griffiths has been found beneficial. Judging from a few instances in which it has been tried, the combination of this solution with carbonate of ammonia, is likely to afford the greatest advantage to patients laboring under some forms of scrophula, that medicine alone can produce. The dose for children, who take it without difficulty, should vary according to the age from twenty drops to one drachm: adults may take from one drachm to three; or even half an ounce if sufficiently diluted.—*Lond. Med. Rev.*

*Cases of Rupture of the Urethra, succeeding a Contusion of the*

* Vide an Experimental Examination of the last edition of the Pharmacopœia Londinensis, by Richard Phillips. Page 99.

*Perinæum,*

*Perinæum, successfully treated.*—I. Harvey, twenty years of age, a man of a sickly aspect, and deformed cast of body, fell from a height, across a ladder, upon the perinæum. Much swelling and tension of the parts from the anus to the scrotum succeeded, with great discoloration. Leeches were applied, and the bleeding encouraged by warm fomentations; after which a poultice was employed, and a saline purgative given, which operated largely during the day. In the evening he took an opiate draught with tartarised antimony, which procured alleviation of his pain, and produced some sleep. The next day he had much tension of the abdomen, and was unable to void his urine. The catheter was introduced without much difficulty, and a large quantity of urine clear and untinged with blood drawn off, and the operation was repeated as often as the patient expressed much uneasiness from distension of the bladder. A similar plan was pursued with little variation for seven or eight days, during which the symptoms were not materially arrested. The discoloration at this time had extended to the scrotum and penis, which were almost buried in the swelling, and which had now become somewhat œdematous. In the night of the eighth day, during a pressing effort to make water, he was seized with a pain of a much more acute kind than what he had before experienced, and on attempting to pass a catheter it met with considerable resistance, and could not now be got into the bladder as usual.

Unavailing attempts were again made to introduce catheters of different sizes and curves, but the obstacle could not be surmounted. The point of the instruments seemed, when it had arrived a little beyond the bulbous part of the urethra, to quit the canal and get into a pouch on the left side of the raphe.

The man's health was now becoming much disordered from constant pain. The pulse was rapid, tongue furred, much thirst, universal heat on the skin, appetite lost and sleep disturbed. It was determined to pass the catheter as far as possible, and to make an incision upon its point, which might be felt externally on the left side of the seam *in perinæo.* This was accordingly done to the extent of more than two inches, through the integuments, much in the same direction as in lithotomy. A large mass of coagulated and grumous blood was pressed out by the fingers, which was followed by a discharge of watery fluid to the extent of a quart at least, of a strong urinous smell, and highly tinged with blood. The catheter might then be distinctly felt and seen, having found its way through the newly formed aperture in the membranous part of the urethra. The patient's sufferings became immediately relieved. A large quantity of water drained off during the night, which reduced the parts nearly to their ordinary dimensions. On the following day, when the dressing was removed, the patient was directed to exert an effort to make water, when the whole contents of the bladder escaped through the wound.

A flexible elastic gum catheter was then introduced, and left in the bladder, through which the man voided his urine, which however, ever, came away partly by the external opening, but chiefly by the catheter, till the wound began to heal. The urine then gradually resumed its natural course, and he perfectly recovered in about a month from the time of the accident, without any other bad symptom.—*Lond. Med. Rev.*

---

*Cases of Ulcers and other Morbid Appearances in the Duodenum, with Changes in the whole Intestinal Tube from previous Disease.* —Mrs. Pratt, æt. 40, complained on Friday March 10th, of pain in her stomach, for which she took some laxative pills. These moved her bowels without affording any relief to the pain. On Sunday she was attacked with chills; violent pain at the scrobiculus cordis, but rather towards the left side; and vomiting of every thing taken into the stomach. The pulse, however, was natural, the skin cool, and pressure upon the part did not aggravate the pain. She thought that relief followed the application of hot flannels.—Monday. Pain and vomiting as before, with hiccough. Pulse 100. Skin hot. Thirst urgent.—Tuesday. The matter vomited had a reddish brown appearance, resembling thin chocolate. Abdomen and epigastric region tense for the first time. Pain and hiccough as before. Pulse 120. Skin hot. Thirst urgent.—Wednesday. Became easy last evening in respect to the pain, but the other symptoms continued, and she died at noon.

Blood was drawn from the arm and from the part affected, by means of cupping glasses, without any abatement of the symptoms. The medicines given were all rejected. No evacuations were obtained although glysters were repeatedly employed.

About eight years ago she labored under ascites, of which she was cured; since that period she has had two severe attacks of dysentery.

*Dissection.*—Extensive adhesions between the peritoneum and omentum, particularly near the os pubis. The omentum appeared very red and of greater length than usual. The small intestines were of a bright red color, and distended to more than twice their usual diameter. The large intestines were for the most part remarkably small and contracted, exhibiting only slight and partial marks of inflammation. On passing the hand between the duodenum and liver, a rupture of the former took place about two inches below the pylorus, and a large quantity of fluid tinged with bile escaped from the intestine. The duodenum was found upon examination to be so thin as to have lost its natural structure: its inner surface was inflamed and marked with distinct ulcerations. This morbid condition extended five or six inches below the pylorus, beyond which the intestine again assumed a more natural appearance. The pyloric portion of the stomach presented upon its inner surface a granulated structure, probably the commencement of ulceration. The pylorus was not at all thickened, but its orifice barely admitted the introduction of the middle finger.

In following the course of the intestines the lower part of the ilium appeared of the natural size, or even smaller, but it was more highly highly inflamed than any other part of the whole canal. The colon at its sigmoid flexure, and the rectum, were smaller than natural, and bound down by firm adhesions.

There was only a very small quantity of feculent matter in the large intestines, and nothing except the fluid above-mentioned in the small ones.—*Lond. Med. Rev.*

---

*Notices of several Important Operations for Aneurism.*—We are glad to find that the ligature of the external iliac artery in cases of inguinal aneurism has at length been practised upon the continent. The history of the following case, in which it was performed by M. Delaporte, one of the principal naval surgeons at Brest, is extracted from the 7th volume of the Mémoires de la Société Médicale d'Emulation de Paris.

Peter Cleck, a marine, 60 years of age, of a good constitution, was admitted into the naval hospital the 11th of August, 1809, for a tumour in the left groin. This tumour had existed eight or ten months, and extended six inches down the thigh, and five transversely from the spine of the ilium to the pubes. The pulsation was visible and the limb was very œdematous. The tumour had increased to a very considerable extent until the 3d of January, 1810, when the operation was performed in the same manner as recommended by Mr. Abernethy and practised by several surgeons in this country. The only difficulty in the operation was the detachment of the artery in attempting to pass the ligatures with Desault's aneurism needle. Two legatures ("faite avec les lacets dont se servent les femmes") were applied, but the vessel was not divided in the interspace. Nine hours after the operation the heat of the left limb was greater than that of the right—the heat and sensibility of both were the same on the following day. The limb was continued in a favourable condition, but a phlegmonous swelling appeared in the loins of the same side on the 5th day—the patient was also feverish, his bowels disordered, and his nights sleepless. The œdema had nearly disappeared, the tumour was stationary but softer—on the 8th day the fever had very much increased, but the limb and tumour were in the most favourable condition, and the inflammation on the loins had disappeared. On the evening of the 11th day the tumour appeared increased in size, it was soft and of a violet colour, but without pain; the wound also had the same appearance. On the 12th day the thigh was cold and discoloured; there were some variations in the tumour, and the pulse was small. On the 13th day the mortification extended to the knee, on the anterior part of the thigh only; the pulse was feeble (capillaire) and there was slight delirium. In the night he died. Upon dissection the abdomen was found to be quite healthy, the lower part of the colon on the left side adhered to the peritoneum, opposite to the wound; behind the peritoneum and in the loins was a collection of matter;—the iliac artery was examined from its origin to the crural arch, the sides were thinner and more dilated than in their natural condition—it was not yet obliterated rated either above or below the ligatures, which were so firm that injection thrown into the inferior part of the aorta, after having tied the primative iliac of the opposite side, did not overcome the resistance which they afforded. The tumour was filled with fœtid clots; the upper end of the artery was under Paupart's ligament. It was at the distance of four inches from the lower, which presented a double orifice by the origin of the profunda and the femoral. The injection entered by the profunda, and was found upon dissection in the femoral artery. The mortification of the limb and the death of this patient unquestionably was not owing to a deficiency of blood after the ligature of the external iliac artery, since the limb lived, the œdema disappeared, and the heat continued natural until the 12th day. M. Delaporte imputes the mortification of the thigh and the death of the patient to the effect of the blood confined in the sac, upon a constitution worn out by the previous fever. We are happy, however, in having it in our power to report the successful result of two other cases in which this operation has recently been performed, the one by Mr. Dorsie, of Philadelphia, and the other by Mr. Kirby, of Dublin. In both cases the operation was perfectly successful.—*Lon. Med. Rev.*

---

*Observations on Hydrophobia.*—Hydrophobia is a disease, which, although not very prevalent in this island, is still sufficiently so to demand the investigation of the medical philosopher. Every year brings with it the account of some few who have died from this afflicting malady. An individual case may only occur to a practitioner in the whole course of his practice; the case is not inserted in any depôt for similar histories; and the opinion of the cause, with the treatment of the disease, both die with the practitioner who made them. Our opinions with regard to the origin of hydrophobia are certainly very vague; for we have one party ascribing the complaint to a specific virus introduced into the system by the bite of a rabid animal; another party, which enjoys an equal number of partizans, denying the influence of a specific virus, and acknowledging the complaint either to have arisen spontaneously, or to have been induced by the power of fear, operating in those habits which are enduced with an irritability of temper, and a certain morbidity of fibre, which are supposed to favor the occurrence of the complaint.

It behoves, therefore, every medical man, to investigate the disease as it affects the animal itself, and to publish to the world a full and accurate detail of those cases of hydrophobia which may have occurred to him; and, when this is considered a moral obligation, we may hope to accumulate a mass of evidence, from which legitimate conclusions may be deduced. In this uncertainty, it might be of use to compare the different conjectures hitherto advanced, which favor one or the other of these opinions, and which would seem to be more required at this present time; as a late publication of Dr. Bosquillon's may make many proselytes to his system. I merely mean to observe in this paper, that, in support of the first opinion, the many cases upon record must induce us to believe, that hydrophobia has has ensued from the bite of a rabid animal; and where not only no suspicion was entertained of the rabies of the animal, but when the mind was confident that no untoward symptoms could ensue, and where, on account of tender age, no terror or *timor hydrophobiæ* could act as exciting causes. I can refer the reader for confirmation of the fact, to the Manchester Memoirs, and to Dr. Ferriar's Medical Facts and Observations. I may mention, in further support, the case of a lady who was bitten by a rabid dog, at Edinburgh, in the year 1806, and, in which case, no terror of future hydrophobia could prove an exciting cause: the symptoms of rabies did not supervene till the termination of eight months from the date of the bite; and during the whole of this period, the patient did not entertain the least idea of that disease which was to terminate her life. The first symptoms (which are not always attending ones) were inflammation and eruption on the part bitten, to which succeeded difficulty of swallowing, and the other symptoms characteristic of the disease. The case of Richard Brown, a boy of seven and a half years old, recorded in the Edinburgh Medical and Surgical Journal for January 1810, likewise militates strongly against that opinion which denies a specific virus as being a cause of the complaint; and Dr. Clarke justly remarks, that "Brown could not have acquired any information on hydrophobia; his age precludes the supposition of reading on the subject; and his relatives were so little prepared, that they did not suspect the nature of the disease." Experiments are wanting to prove in what manner the disease of rabies is communicated from the dog; whether the cause is in a vitiated state of the saliva, or derived from any peculiar poison which may be secreted, as we observe in venomous serpents; but proofs are not wanting to shew, that the disease *has* arisen from the influence of the bite, and that the opinion of Dr. Bosquillon is erroneous. Dr. B. acknowledges only three causes for the complaint, viz. 1. Inflammation of an internal viscus; 2. Lesion of a nerve or tendon; and, 3. Fear, which latter is the most common cause of all. Dr. B. says, (Vide L'Esprit des Journaux, Tome II. Fevrier 1809,) "Elle (La Terreur) a particulierèment lieu à la suite de la morsure des chiens pretendus, ou réellement enragés. Elle ne peut être qu'une suite de nos préjugés; car tout remede capable de rassurer l'imagination frappée en arrête avec certitude les progrès. Peut on avoir *des preuves plus certaines qu'elle n'est pas due à un virus particulier.*"

The Doctor wishes to prove, from premises that will not bear him out in his conclusion, that hydrophobia cannot ensue from the bite of a dog. His cases only shews that rabies, or a disease very similar, may be induced by other causes. In those cases which he mentions, the dogs were not proved not to have been mad; the non-existence of a specific virus could only be suspected, as their diseases were not attended to. In the paragraph, however, above stated, the exemption from rabies must have been certain; as it is not to be supposed, that any impression made upon the mind, could stop the progress of genuine rabies produced by specific virus. The case of the child, recorded in the same Journal, only proved the instance of a child a child dying through fear, in which some anomalous symptoms were conspicuous. Nevertheless, we must allow that hydrophobia, or a disease very similar to it, has in very many instances arisen spontaneously, or been induced by powerful impressions upon the mind in nervous habits, as we cannot suppose that so active a virus can exist for many years in the system, without producing its effects. In this opinion I heartily agree with Dr. Bosquillon, but differ from him, unless he disallows of the possibility of the disease being communicated from the canine to the human species. *Edin. Jour.*

---

THE LATE PHÆNOMENON AT BARBADOES.

" *Buttall's Plantation, Parish of St. George, May* 2, 1812.

" I hasten to give you some account of a most awful visitation of Providence which took place yesterday in this neighbourhood, and I believe generally throughout this island.

" Early in the morning, as we imagined from its darkness, my wife requested me to view the sky, which had a very odd appearance. Upon looking at my watch, we were very much surprised to find it was so late as half past six o'clock, A.M. which neither of us could credit, until, on comparing it with her's, they were found to agree.

" In the north-east, in which the sun ought to have been seen, as it had been up three quarters of an hour, a very large and dense cloud of ferruginous color, and at no great height above the surface, obscured the firmament, but in such a manner that the trees and shrubs in the garden, and the country to the north and east of our house, presented the same lights and shadows as they usually do when the moon is sometimes bright and sometimes dark in the alternation.

" Another cloud, not quite so dense, of a dusky blue color, and about the same height, hung over the edges of the whole southern horizon, under which the sky appeared of a silvery color, from which issued a very shining light, by which objects to the northward, when our backs were turned to the light, were seen as distinctly, if not more so, as at noon-day; while objects between us and the light were scarcely perceptible.

" Above these clouds, and in every other part of the sky which was not occupied by the ferruginous clouds, were other clouds of a whitish grey color, which were carried over the islands with great velocity from the north-east, in which direction the wind blew the whole day with very little variation; although under them there was not a leaf stirring, or breath of air moving.

" A solemn and unusual stillness pervaded every place, now and then interrupted by the sound of negroes at work with their hoes, which the surrounding silence seemed to augment.

" Forcibly struck with all these appearances, I could not help expressing to Mrs. D. my apprehensions that this island was about to be visited with some dreadful commotion; and, as our house was on the acclivity of a hill, about sixty feet above the level of the works, at which our friends Mr. and Mrs. H. resided, I thought it advisable we should join them; which we did without delay.

"By the time we reached the works it was seven o'clock; and, as the darkness began to increase considerably, all the negroes were recalled from the fields and ordered to their houses, where most of them went to bed with much indifference, considering the darkness only as an early night.

"At half past seven o'clock it was so dark that candles were brought in. At eight o'clock it was pitch dark in the open air; or, in other words, so dark that we could not perceive our hands when held up before our faces at two feet distance. No night at home in winter, when neither the moon nor a star is to be seen, was ever more sombre. This darkness continued of the same intenseness until 25 minutes past twelve o'clock; that is, for the space of four hours and 25 minutes, at which time we perceived very indistinctly the outlines of large and near objects. At half past twelve o'clock we distinguished them more correctly, from which period the light increased until between three and four o'clock, P.M. but was very obscure.

"From the time at which I got up in the morning, until we went to bed at home in the evening, at eight o'clock, there was a constant fall from the clouds of a substance in extremely fine flakes, which, when first gathered from our clothes, had the appearance of the dust of wood-ashes; but which, when suffered to accumulate, assumed the resemblance of powdered rotten-stone, and possessed the same quality of cleaning brass.

"In order to ascertain the quantity which had fallen, Mr. H. last night took up that which lay upon a foot square, when it measured three pints, somewhat pressed into the measure, and weighed one pound and three quarters.

"This morning another square foot, where the surface was hard and level, gave, in five-eighths and one-half of an inch in depth, three pints loosely filled up in measure, and one pound seven ounces and a half in weight.

"Against the bottom of windows, doors, and walls, it was considerably deeper. But, assuming the product of my experiment as the medium quantity which fell on a foot square throughout the island, and estimating from our best maps the quantity of land in the island at 106,470 acres,* the total quantity of this extraneous substance which is now on its surface, independent of that which is upon the trees, could not be less than 1,739,187,750 gallons, wine measure,† or 6,811,817,512 pounds avoirdupois.‡

| | Acres. | | Acres. | | Acres. |
|---|---|---|---|---|---|
| * Christchurch | | St. George .. | 10,795 | St. Joseph . . . | 6,010 |
| parish . . . . | 14,310 | St. John . . . . | 8,600 | St. Andrew . . | 8,780 |
| St. Philip . . . | 15,040 | St. James . . . | 7,800 | St. Peter. . . . . | 8,330 |
| St. Michael . . | 9,580 | St. Thomas . | 8,500 | St. Lucy . . . . . | 8,725 |

† 106,470 acres in the island, multiplied by 43,560 square feet in one acre, is equal to 4,637,833,200 square feet, multiplied by 3 pints per foot, is equal to 13,913,499,600 pints, divided by 8 pints in a gallon, is equal to 1,739,187,450 gallons, wine measure.

‡ 4,637,833,200 square feet in the island, multiplied by 1lb. per inch, 7½ c. feet is equal to 6,811,817,512lbs. avoirdupois.

"The

"The fall of this substance was least in the morning and evening, and greatest between nine and twelve o'clock. During this last period, when any of us went out into the air, we perceived a smell similar to that which arises from water thrown upon hot embers, but with no increase of heat, to the best of my perception: on the contrary, according to my own sensations, it was rather colder during the continuance of this phænomenon than in common.

"I had unluckily left my thermometer behind me, and did not get it until one o'clock, P.M. at which time I hung it in a gallery facing the south, and open to the air, when it settled at $77\frac{1}{2}$, and continued at that height until five o'clock in the afternoon, after which I forgot to examine it.

"All those who ventured out with lanterns during the darkness reported they had heard strange noises and cries in the air; but, as they also added, that these noises and cries had followed them, and that objects had been seen or felt flitting past them, it was no difficult matter to convince most of them that these sounds arose from the birds and bats, which the lights they carried with them had attracted. Many animals, which had been loose at the commencement of the darkness, found their way to the lights in the houses, and remained at the house during the continuance of the darkness.

"Some land birds flew into the rooms where the windows were open, and some few sea birds were known from their cries to be hovering about the buildings.

"After this dismal scene had continued some time, my apprehensions were considerably lessened, by bringing to my remembrance the account of the younger Pliny of the eruption of Vesuvius; and the fact mentioned to me by yourself, of a substance similar in some degree to what has fallen here, having been gathered off the sails of a ship, at a great distance from any known land; from whence I began to hope, that as no cinders had made their appearance, the phænomenon, which was alarming us, might be the effect of some distant volcanic eruption, and not the fore-runner of any more dreadful visitation than that which was existing at the moment.

"This idea has been strengthened by the following circumstances:

"About one o'clock in the morning Mr. H., and several others on the plantation, heard a very heavy and quick firing, neither as minute guns from a ship in distress, not in continuance as from ships engaged, but in peals at intervals, from the southward. The same firing was heard so distinctly in town and its vicinity, but in a westerly or north-westerly direction, that it is said our Governor, who is also commander of the forces in the leeward island, repaired about two o'clock to the garrison, which was kept under arms all the night, from a surmise that Admiral Sir Francis Laforey, who had sailed to the northward the evening before at sun-set, with the Dragon, and a tender only, had fallen in with the enemy's fleet of four sail of the line and four frigates, which, by accounts from Madeira, are reported to have passed that island.

"As

"As nothing has transpired this forenoon to countenance either the supposition of an engagement, or of a ship having been lost last night off our coast, it is very possible, and by no means improbable, that the noise in question may have proceeded from a volcanic eruption, which, without any earthquake having been perceptible in the island, may have produced those phenomena which I have attempted to delineate.

"During last night the weather has been rather calm than otherwise, and this morning the appearance of the country resembles the land in the neighbourhood of the river Nith, near its entrance into the Solway Frith, when it has been overflown by a high tide. Many shrubs and low trees with spreading branches have been split by the weight of the substance which has fallen on them, and the sugar-canes are bent down to the ground.

"On removing the substance from the surface, the grass continues as green at least as it was before, without any appearance of being scorched.

"The thermometer this day, when placed in a room open to the air, but not exposed to the direct or reflected rays of the sun, has varied from 70 to 80, which is three degrees higher than it was yesterday at the same hour of the day.

"Being now relieved from the darkness and its concomitant fears, we are beginning to distress ourselves about the effect which the fallen substance is likely to produce on the fertility of the land: to ascertain which, with as little delay as possible, my father-in-law and I have this morning planted various seeds, useful and ornamental, in this substance singly, and also in it when combined with different proportions of the soil; trusting that the same good Providence, which has carried us in safety through the danger of yesterday, may be pleased to render this visitation a blessing, and not a curse, to the island.

"Lest you may wish to ascertain the component parts of this substance, I will send you, by the first good opportunity, the portion of it I gathered from the surface of a foot square, as I have already noticed, and in the interim I inclose you a few grains."—*Phil. Mag.*

METEO-

